# Mucosal Barrier in Ulcerative Colitis and Crohn's Disease

**DOI:** 10.1155/2013/431231

**Published:** 2013-05-07

**Authors:** A. E. Dorofeyev, I. V. Vasilenko, O. A. Rassokhina, R. B. Kondratiuk

**Affiliations:** National Medical University, Donetsk 83003, Ukraine

## Abstract

*Background*. The mucus layer in the gastrointestinal tract plays important role in host innate defense, regulation of secretion, and absorption processes, maintaining colonization resistance, which composes the integrity of protective mucus barrier in the large intestine. Investigations of mucin expression in the colon mucosa can improve the understanding of protective function of mucosal barrier in ulcerative colitis (UC) and Crohn's disease (CD). *Materials and Methods*. 77 patients with UC and CD were examined. Histological analysis of colon mucosa was done by standard method (haematoxylin-eosin, alcian blue at pH 1.0 and 2.5 to determine sulfated and nonsulfated glycosaminoglycans and glycoproteins, and goblet cells). To characterize the mucus production the PAS-reaction was performed. Immunohistochemistry was performed using monoclonal mouse antibodies raised against MUC2, MUC3, MUC4, and TFF3 (USBiological, USA). *Results*. The moderate expression of MUC2 and MUC3 (50.0% and 32.1%, *P* = 0.03) and high expression of MUC4 and TFF3 in the colon mucosa were observed in all patients with CD. The intensive labeling of MUC4 and TFF3 occurred more often (42.9% and 57.1%, *P* = 0.03) in patients with CD. The level of expression of secretory MUC2 and transmembrane MUC3 and MUC4 in all patients with UC was low, up to its complete absence (59.2% and 53.1% cases, *P* = 0.05). TFF3 expression had high and medium staining intensity in patients with UC. *Conclusions*. Different types of mucins synthesis, secretion, and expression were found in patients with UC and CD. The expression of mucin MUC2, MUC3, MUC4, and TFF3 correlated with the activity of disease and the extent of the inflammatory process in the large intestine. The most pronounced alteration of mucins expression was observed in patients with severe UC and CD.

## 1. Introduction

Protective function of the intestinal mucosal barrier depends on the coordinated regulation of the mucus layer, epithelial cells, host innate, and adaptive immune response [1, 2]. The mucus layer provides the first line of defense against xenobiotics, microbes, viruses, fungi, protozoa, and so forth, [2, 3]. Epithelial mucins is a large group of secreted and transmembrane glycoproteins reached with amino acids consequences of serine, threonine, and proline and associated with numerous of oligosaccharide chains, which form gel-like structure [2–4]. More than 20 mucin genes (MUC) have been identified [4, 5]. The most genes of secretory mucins are located on chromosome 11.p15.5 [1, 4, 6]. The level of expression and the degree of glycosylation of mucins characterize variability of the protective function of mucins.

 Goblet cells produce secretory (MUC2, MUC5AC, MUC5B, and MUC6), membrane-associated mucins (MUC1, MUC3, MUC4, MUC13, and/or MUC17), and trefoil factors (TFF1-3), which are responsible for epithelial restitution [2, 7]. MUC2 is the major mucin secreted in the large intestine [3, 6]. MUC1, MUC3, and MUC4 are mostly expressed in the small intestine but also can be found in the apical membrane of goblet cells in the colon [1, 2, 7]. Mucins are secreted by two pathways: basal and stimulated secretion in response to bioactive or epigenetic factors such as microbes, toxins, pro-inflammatory cytokines, neuropeptides, and growth factors [3, 8]. These factors lead to change of the expression of mucins in response to inflammation in the colon mucosa. Affected mucus barrier increases permeability for bacteria, microbial products, and toxins that lead to damage of epithelial cells and result in systemic inflammatory process evident in patients with ulcerative colitis (UC) [8–10]. At the same time, hyperproduction of mucins and abnormal glycosylation is typical for Crohn's disease (CD) [11–13]. It still remains unclear if the differences in mucin production are the cause or the result of different immune response in patients with inflammatory bowel diseases (IBD). Investigations of mucins in the colon mucosa can improve the understanding of the role of mucins in maintaining the integrity of protective mucosal barrier in UC and CD.

### 1.1. Aim of the Study

The aim of the study was to analyze expression of mucins (MUC2, MUC3, and MUC4) and trefoil factor-3 (TFF3) and its influence on colon mucosal barrier in patients with ulcerative colitis and Crohn's disease. 

## 2. Materials and Methods

77 IBD patients (39 women and 38 men) were examined in acute phase. Average age was 38.3 ± 9.2 years. Diagnosis of UC was based on clinical symptoms, endoskopy, X-ray examination, and histological findings. Patients with CD and UC were classified according to Montreal classification [14]. Clinical severity of UC was based on Mayo score assessment [14, 15]. The activity of CD was measured by Crohn's disease activity index (CDAI) [15, 16]. Endoscopic examinations were carried out with visual examination of the colon mucosa and assessment of endoscopic index (EI) [16]. Bioptates of colon mucosa were stained by haematoxylineosin and alcian blue at pH 1.0 and 2.5 to determine sulfated and nonsulfated glucosaminoglycans and glycoproteins and goblet cells in the colon mucus layer. To characterize the mucus production the PAS reaction was performed.

### 2.1. PAS/Alcian Blue Staining

Bioptates were fixed in 5% formalin solution. Dewaxed sections were immersed in ethanol in growing concentrations 50%, 60%, 70%, 80%, and 100% for 5 minutes in each solution. Then bioptates were placed in an ortoxylol solution during 20 minutes. The slides were processed by ortoxelol and ethanol on descending concentration, then oxidized in 1% periodic acid in water at room temperature for 10 min, washed in water for 5 min, and stained by haematoxylin-eosin or Shiff's solution. The stains were prepared on a buffer solution of an acetic acid pH 2.6 and 0.1 normal solution of a hydrochloric acid pH 1.0 for staining by alcian blue. The number and maturity of goblet cells, as well as the content of mucus in them, the maturity of mucus, the intensity of cell infiltrations, and their character, were determined by histological assessment.

### 2.2. Antibodies

Immunohistochemistry was performed using monoclonal mouse antibodies raised against MUC2, MUC3, MUC4, and TFF3 (USBiological, USA).

### 2.3. Immunohistochemistry (MUC2–4, TFF3)

Bioptates were fixed in 10% formalin solution (pH 7.2–7.4) for 18–24 hrs with subsequent embedding in paraffin blocks. Paraffin tissue sections (4 *μ*m) were dewaxed and rehydrated. Antigen retrieval used for the detection of the monoclonal antibodies was done by 10 mM citric acid and pH 6.0 at 95°C for 20 min. The sections were washed subsequently in 0.15 M NaCl and 0.1 M Tris/HCl buffer (pH 7.4) containing 0.05% Tween-20. Nonspecific binding was blocked by protein block (Dako) for 30 min. The sections were incubated with the BC2 antibody diluted in Antibody Diluent for 1 h, and then incubated with Broad Spectrum Poly HRP Conjugate (USBiological, USA) for 30 min in 37°C and then with 3.3-diaminobenzidine for 30 min. The sections were counterstained with haematoxylin for 30 sec. To receive blue-colored effect 37 mmol clonal antigen's assessment was compared with a negative control.

### 2.4. Scoring

The intencity of Muc 2-4, TFF3 staining was evaluated in score points, which were classified by four rates: absence of staining (<1% of stained cells, score = 0); low level of staining (1–30%, score = 1); medium level of staining (30–80%, score = 2); high level of staining (up to 80% stained cells, score = 3). The scoring of the staining was performed by an individual blinded assessment of morphologist. 

### 2.5. Statistics

Individual data points were presented. For all statistical analyses, differences were considered as significant for *P* ≤ 0.05 and nonparametric Mann-Whitney *U* test was applied to ascertain differences. All statistics were done using MedStat and Statistica 6.1 (StatSoft).

## 3. Results

### 3.1. Crohn's Disease

The most of patients with CD, 25 (89.3%), had established diagnosis between 17 and 40 years (A2), and 3 (10.7%) patients had A3. Patients with ileal or upper gastrointestinal involvement were not included in the study to receive homogeneous group for analysis. Colonic location (L2) of CD predominated, 19 (67.9%) patients, ileocolonic (L3) extension was observed in 9 (32.1%) patients. Nonstricturing, nonpenetrating behaviour of disease (B1) prevailed, 20 (71.4%) patients; strictures (B2) were found in 3 (10.7%), and penetrations (B3) only in 5 (17.9%) CD patients. Perianal involvement did not occur in the investigated group.

 Minimal activity of CD was observed in 9 (32.1%), moderate activity—in 15 (53.6%) patients and severe CD in 4 (14.3%) patients. Total CDAI in CD group was equal to 283.6 ± 18.2 score points. EI was 1.9 ± 0.7 in all patients with CD.

 Histological examination of the biopsies of patients with Crohn's disease revealed inflammatory ulceration of colon mucosa with moderate epithelial damage and structural changes. Transmural lymphocytic infiltration with focal lymphoid hyperplasia and fibrosis of the mucosal layers was noted in all cases. The specific feature was accumulation of lymphocytes with formation of lymphoid follicles, in 19 (67.8%) patients, combined with diffuse infiltration by neutrophils and macrophages. The number of goblet cells did not change significantly. Goblet cell hyperplasia was observed in 17 (60.7%) patients. General mucus production was not reduced. At the same time, in mucosa adjacent to the ulcerations depletion of the mucus production was noted. Transmural ulcers were revealed in 6 (21.4%) cases. Ulceration associated cell lineage (UACL) was observed in 7 (25.0%) CD patients. Epithelioid granulomas in the submucosal layer were found in 11 (39.3%) patients with CD. The PAS-reaction was moderate in the most of patients with CD, 22 (32.7%). Alcian blue at pH 1.0 revealed intensive staining in 20 (71.4%) patients; moderate intensity was noted in 8 (18.4%) patients, moreover, at pH 2.5 a moderate and intensive staining was found in 18 (64.3%) patients. These findings indicate imbalance in glycosylation with prevalence of weakly sulphated mucins in patients with CD.

**Figure 1:**
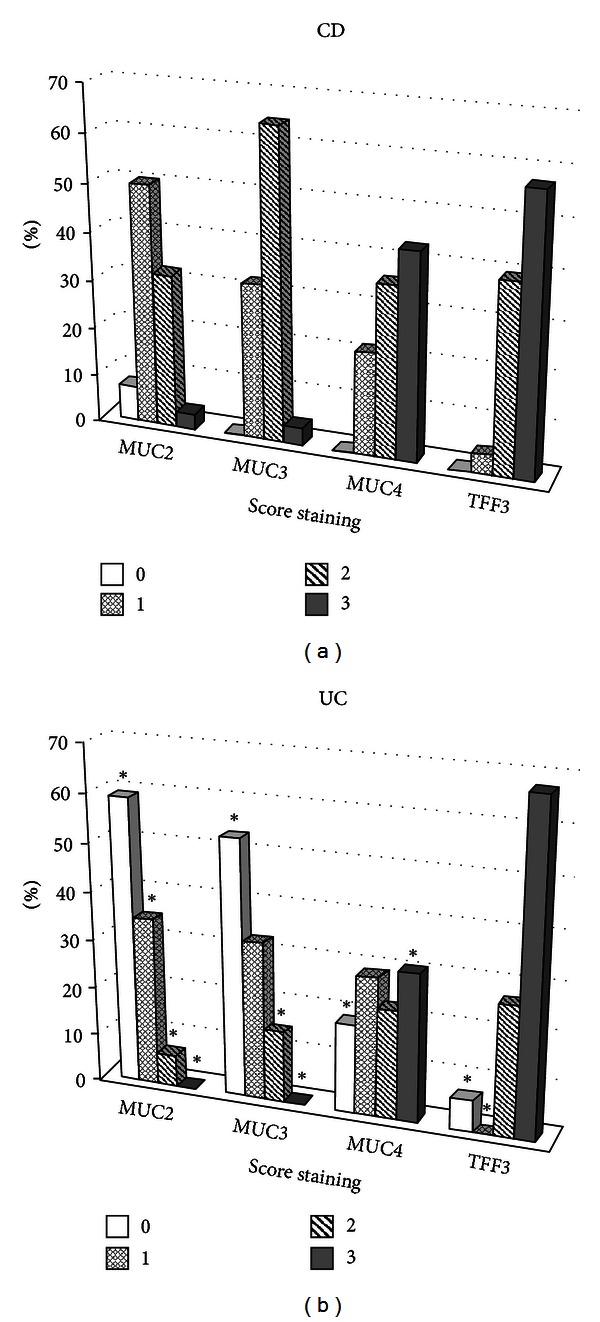
General mucins (MUC2-4) and TFF3 expression in CD (a) and UC (b) patients. Histological score of the staining intensity from 0 to 3 ((b) *P ≤ 0.05 compared to CD, Mann Whitney *U* test).

 The moderate expression of MUC2 and MUC3 and high expression of MUC4 and TFF3 in the colon mucosa were observed in all patients with CD. However, the distribution and intensity of the labeling were heterogeneous, which are commonly accompanied with CD. Mild and moderate intensities of the staining were noted for MUC2 (50.0% and 32.1%, *P* = 0.03) cases, respectively. For MUC3 low labeling was observed in 9 (32.1%) and moderate in 18 (64.3%) patients. High intensity for both MUC2 and MUC3 was not above 10% of CD patients (Figure 1(a)). The tendency for colocalization and features of MUC4 and TFF3 expression have been noted. Moderate intensity of the staining of MUC4 and TFF3 was detected in 35.7% and 39.3% cases, respectively. The intensive labeling of MUC4 and TFF3 occurred more often (42.9% and 57.1%, *P* = 0.03) in patients with CD. At the same time, the differences of mucins disposition depended on involved epithelial structures. Disappearance of MUC2 and MUC3 near the UACL areas was found, which indicates acute stage of disease and prevalence of inflammatory processes in the mucosa instead of reparation. Whereas, strong signals of MUC4 and TFF3 were observed in the surrounding mucosa of goblet cells. Intensive labeling prevailed especially in the bottom of the goblet cells and was present in cytoplasm. At the same time the intensity of the signal decreased from the basal to the surface epitelium and to the apical part of the ducts. 

 Mucins expression in patients with CD varied dependening on the clinical activity of disease. Patients with mild severity of CD had moderate and intensive expression of MUC2 in basal part of goblet cells and in cytoplasm in 15 (53.6%) of cases. Most of the patients, 19 (67.80%), had moderate expression of MUC3 around vacuoles of goblet cells and in stromal epithelium (*P* = 0.01). MUC4 and TFF3 had strong staining in crypts epithelium, but were not determined in stroma.

 In moderate CD weak staining of MUC2 and moderate staining of MUC3 in vacuoles were observed in 11 (73.3%) and 9 (60.0%) patients, respectively (*P* = 0.05). At the same time, mild expression of these mucins was found in stromal epithelium. Level of MUC4 varied from low to high labeling, 6 (40.0%), in periphery of vacuoles and in the basal part of cytoplasm. TFF3 expression was moderate and high in surface epithelium in the most of the patients, 25 (89.3%).

In patients with severe CD the quantity of goblet cells and the expression of MUC2 were accompanied and significantly increased in surface epithelium and in stroma. In the surface epithelium moderate staining of MUC2 and MUC3 was found almost in all patients (up to 70% of cases). The level of MUC3 expression varied from moderate to intensive in the edge of vacuoles. Epithelial proliferation in the basal part of crypts and mild proliferation of stroma were identified with Muc3. Slight signaling for MUC4 stained goblet cells and surface epithelium and was absent in stroma in all patients. TFF3 expression was high in crypts and goblet cells (75% of patients) and was not present in stroma.

### 3.2. Ulcerative Colitis

11 (22.4%) patients had distal UC (E1), 32 (65.3%) persons suffered from left-sided UC (E2) and 6 (12.3%) patients had pancolitis (E3). Patients with moderate severity of UC predominated among all UC patients, 28 (57.1%) persons. Mild severity of disease occurred in 16 (32.6%) patients. Only 3 (10.3%) patients had severe UC. Index Mayo in all UC patients consisted of 2.6 ± 0.9 score points. Severity of UC correlated with extensive character of inflammation in the large intestine. EI was equal to 2.1 ± 0.5 in all patients with UC.

 The histological analysis of the sections showed increased infiltration in colon mucosa; but reduced quantity and depletion of goblet cells, which were found in all patients with UC. Reduction of goblet cells correlated with severity and the extension of disease. Patients with mild and moderate UC had moderate depletion of goblet cells with small vacuoles and low content of mucus (62.5% and 71.4%, resp.). Patients with severe UC had almost total depletion of goblet cells and instant decrease in their quantity. Moreover, significant qualitative mucus modification in UC patients has been identified. The PAS-reaction showed a moderate level of PAS-positive substances in 16 (32.7%) patients, while decreased PAS-reaction was found in 33 (67.3%) of UC patients. That indicates a significant depletion of mucus production and abnormal glycosylation which decrease the protective function of mucus layer in UC. Active leucocytes containing glycogen were found in the most of UC patients, 39 (79.6%). Alcian blue at pH 1.0 revealed weak staining in 40 (81.6%) patients; moderate intensity was noted in 9 (18.4%) patients. However, at pH 2.5 moderate and intensive staining was found in 31 (63.2%) patients. At the same time, in 18 (36.8%) patients mild staining was observed. These findings indicate imbalance between sulfated and unsulfated glucosaminoglycans that results in the decreasing of protective function of the mucins in patients with UC. UACL was observed in 27 (55.1%) patients with UC (*P* = 0.03).

The level of expression of secretory MUC2 and transmembrane MUC3 and MUC4 in all patients with UC was low. However, the most decreased expression was revealed for MUC2 and MUC3, which manifested as weak staining vacuoles of goblet cells (34.7% and 32.6% cases, respectively, *P* = 0.03) up to its complete absence (59.2% and 53.1% cases, *P* = 0.05). The strong signaling was found only in 3 (6.1%) patient with UC in the cytoplasm around the mucin vacuoles and moderate staining in the basal epitelial cells. At the same time, low expression of MUC2 and MUC3 in the surface epithelium, was found in the most of patients, 35 (73.5%), *P* = 0.01 (Figure 1(b)). In 29 (59.2%) UC patients staining of stromal cells varied from low to high staining of MUC2 and MUC3. MUC4 level was also low in the most of patients with UC, but decreased lesser compared with MUC2 and MUC3. Moderate level was identified in 11 (22.4%) and intensive labeling in 15 (30.6%) patients with UC (*P* = 0.03). TFF3 expression in the colon mucosa in patients with UC was high in contrast to the secretory mucins: 33 (67.4%) of patients had high and 13 (26.5%) medium staining intensity. Moreover, the localization of TFF3 was also inhomogeneous: more expression was observed in the basal cells of the crypts, moderate staining in the vacuoles of goblet cells. In some cases a marked surface localization of TFF3 in combination with the absence or weak expression of MUC2 and MUC3 was found.

**Figure 2:**
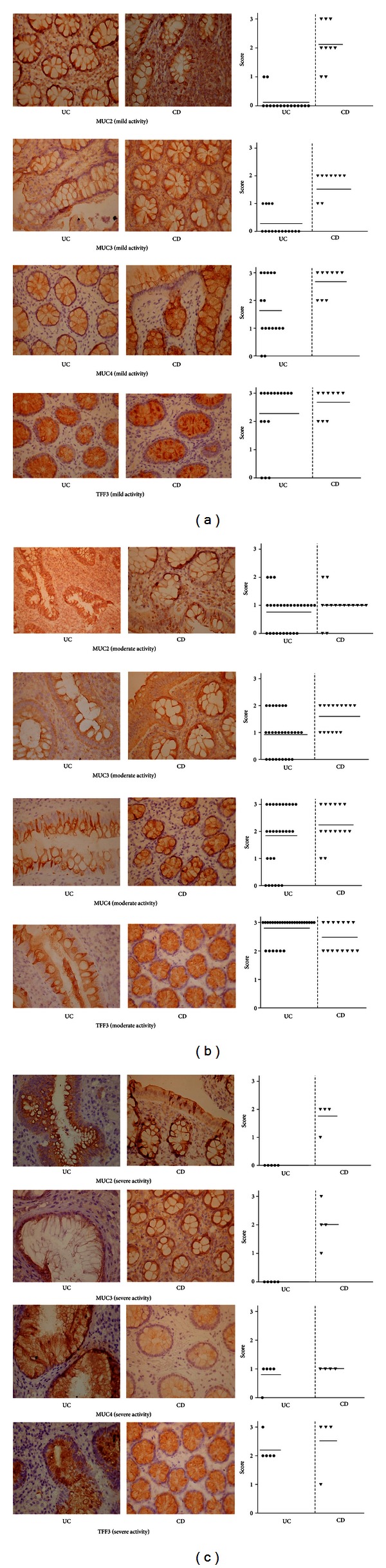
Mucins (MUC2-4) and TFF3 expression depending on severity of CD and UC. The apical surface of the large intestinal epithelium, crypts, goblet cells, and stromal epithelium are lined by the secretory mucin MUC2 and transmembrane MUC3, MUC4, and TFF3 (brown colored). Alcian blue (1.0 and 2.5) positive staining structures (blue, highly glycosylated; purple, medium glycosylated). The photographs are displayed in 400x magnification.

 The intensity of the identified changes in mucins expression varied depending on the severity of ulcerative colitis. Thus, in patients with mild activity of UC significant violations of MUC2 expression were determined (Figure 2). MUC was not detected in 14 (87.5%) of patients. MUC3 was detected only in 4 (25.0%) UC patients. At the same time, mild expression was observed for MUC4 in 7 (43.7%) and was intensive in 5 (31.2%) patients.

 The differences of MUC2 and MUC3 expression between patients with mild and moderate UC were not significant. In patients with moderate UC the level of secretory MUC2 expression was detected in 18 (64.3%) patients. The most of patients 15 (53.6%) had weak staining of mucin. The membrane-bound MUC3 was detected in 67.8% cases and the expression of mucin was minimal—12 (42.8%) patients. At the same time, medium labeling of MUC3 was revealed in 7 (25.0%) of UC patients. More severe violations of MUC4 expression was observed in patients with moderate UC: absence of MUC4 occurred in 6 (21.4%) patients, moderate staining in 9 (32.1%), and high in 10 (35.7%) patients with UC. The strong labeling of TFF3 was also prevailed patients with moderate UC—22 (78.5%) cases (*P* = 0.01).

 In patients with severe ulcerative colitis an absolute deficiency of expression of MUC2 and MUC3 in goblet cells were observed in 100% of patients, indicating the most pronounced changes in the synthesis and secretion of mucins in patients with severe UC. The expression of MUC4 was observed in 80% of patients; however, it was low. At the same time, level of TFF3 remained moderate in 80% of patients, but decreased comparing with moderate severity of UC. 

## 4. Discussion

The total level of expression and glycosylation of mucins in patients with UC was low which indicates significant decrease in secretory activity of the colon mucosa reducing its protective function in UC and characterizes an inefficiency of repair process in mucosal barrier of the large intestine during exacerbation of ulcerative colitis. In contrast, patients with CD had moderate or increased expression of secretory and membrane-bound mucins that was accompanied with increased sulphation of mucins and thickness of gel layer in the colon mucosa in Crohn's disease.

 The reduction of goblet cells, decrease in glycosylation of mucins, absence of mucins MUC2, and MUC3 expression in goblet cells were found in affected colon mucosa in patients with UC. Whereas, intensive staining of mucins in the cytoplasm around the vacuoles and in the basal epithelial cells, which was found in UC and CD, may reflect not only insufficiency of mucus production, but also high intensity of the metabolic processes and proliferation in the epithelium of the large intestine. More than half of patients with UC had heterogeneous staining of stromal cells from low to high expression of MUC2 and MUC3, which testifies to the active mitotic and differentiation processes of epithelial cells into stromal goblet exocrinocytes. At the same time, general expression of MUC2 and MUC3 was moderate in goblet cells and in vacuoles in CD patients. MUC2 appears in two forms: mature, as a secretory product of goblet cells and immature, in secretory granules of cells that are not phenotypically goblet cells [5, 17]. Thus, mucus production in CD increased due to mature and immature MUC2 in goblet cells, and intensive proliferative processes with immature MUC2 and MUC3 revealed in stroma. These changes can be assumed as a compensatory reaction induced by the inflammatory process in the large intestine or may contribute to CD or UC by different types of affection of the mucus barrier. The expression of secretory mucins is induced by proinflammatory cytokines: TNF-*α*, INF-*γ*, and IL-6 [4, 18]. Therefore, absence of expression in the vacuoles of goblet cells and its appearance in the stroma confirm depletion of goblet cells and localization of inflammatory process in the lamina propria of colon mucosa, which is typical for UC. Whereas, hyperplasia of goblet cells increased mucins production with high viscosity, moderate expression of MUC2 and MUC3 was specific for patients with CD. 

 At the same time, chronic inflammation caused by bacterial infections (haemolysing and enteropathogenic *E. coli*, *Salmonella enteritidis*, *Clostridium difficile* etc.), associated with high expression of secretory mucins and its hyperproduction with thickening of the outer mucosal layer [3, 4, 19]. This process was induced by binding of specific pathogen-associated molecular patterns receptors (NOD2/CARD15) and toll-like receptors (TLRs) that protect mucosa from microbial and parasites invasion [2, 20]. Mutation of NOD2/CARD15 and TLRs more often occurs in patients with Crohn's disease and confirms the hypothesis about the role of infections in affected innate immune response and intensive mucus production as features of pathogenesis of CD. Our patient with UC had significant reduction of the MUC2, MUC3 expression, and mucins production in comparing with CD patients that can be additional differential criteria for these two inflammatory bowel diseases. 

 The levels of MUC4 and TFF3 expression were compared to high MUC2 and MUC3 in patients with UC that may indicates minimal preservation of the inner layer of mucus by MUC4. These changes may represent a nonspecific reparative process in the large intestine to compensate injured mucosal barrier. On the other hand, patients with CD had increased level of expression of MUC4 and TFF3 almost in all the stages of disease. An excessive activation of the transmembrane mucins leads to stimulation of nuclear factor-*κ*B and canonical inhibitor of nuclear factor-*κ*B kinase-*β*, associated with neoplasia induced by chronic inflammation [2, 3, 21], which may explain the high incidence of malignancy with prolonged duration of IBD. A combination of TFFs and mucins provides increased protective properties of mucosal barrier in the large intestine. TFFs increase the viscosity of the mucus layer and protection from microbial fermentation of mucins [4, 22]. Moreover, combinations of TFFs and mucins facilitate restitution in epithelial wound healing [22]. In normal colon mucosa colocalization for MUC2 and TFF3 is typical [1, 4]. Patients with UC had imbalance of these two main products of goblet cells, which may reflect a violation of regulatory effects of trefoil peptides on the expression and protective properties of mucins. In contrast, patients with CD had excessive expression of MUC4 and TFF3 that may induce intensive mucus production and aberrant upregulation of mucins synthesis with imbalance of mucosal barrier function.

 Negative correlation for mucins expression depending on the severity of ulcerative colitis was revealed. Decreased MUC2 and MUC3 expression was found in all stages of UC. But the most pronounced changes in the synthesis and secretion of mucins were observed in patients with severe UC. In patients with mild activity of UC only MUC4 and TFF3 had high level of expression, while MUC2 and MUC3 were significantly decreased. This evidence probably indicates a compensatory reaction in the increasing of the synthesis of membrane-associated mucin MUC4 and TFF3 instead of reduction of overall secretory activity.

 In addition to depletion of goblet cells and decreasing of MUC2 and MUC3, expression of MUC4 in moderate UC was also lower in comparison with mild UC. Only level of TFF3 remained high. This reflects more significant changes in the injured colon mucosa in moderate UC and devastation of compensatory mechanism of the mucosal healing. 

 Alteration in the types of mucins and significant effect on the mucus barrier function were noted in patients with severe UC. This revealed “unresponded” goblet cells to the stimulating effect of pro-inflammatory cytokines in acute phase on the expression and secretion of mucins due to long duration of disease and exhaustion of compensatory protective mechanisms of the mucus layer in UC. Even high expression of TFF3, which was revealed in most of the patients with UC in mild and moderate severity of UC, decreased to moderate level in severe stage of UC.

In contrast to UC mucin expression in patients with CD increased with gradation of the disease activity. In mild severity of CD it varied from moderate to intensive expression for MUC2, MUC3, MUC4, and TFF3 as in goblet cells and secretory granules, as in stromal epithelium that revealed substantial differences from UC. In moderate CD low staining of MUC2 was observed, which indicates decreasing of mucus secretion and prevalence of proliferative processes. At the same time, the levels of transmembrane MUC3 and MUC4 remained moderate in most of the patients in crypts epithelium and in stroma. The most significant changes in the colon mucosa were found in patients with severe CD. The expression of MUC2 and MUC3 was accompanied with goblet cells hyperplasia, and was high in surface epithelium and in stroma. In contrast, level of membrane-bound MUC4 was decreased comparing with mild and moderate CD, which reflects the most profound changes of mucus synthesis with imbalance of secretory and membrane-associated mucins in CD. High expression of TFF3 was found in all CD patients, and, probably, was not accompanied with the stage of disease, but reflects effect of the general mechanisms of mucins synthesis in CD. 

## 5. Conclusions

Different types of mucins synthesis, secretion, and expression were found in patients with ulcerative colitis and Crohn's disease. The expression of mucin MUC2, MUC3, MUC4, and TFF3 correlated with the activity of disease and the extent of the inflammatory process in the large intestine. The most pronounced alteration of mucins expression was observed in patients with severe UC and CD. Revealed changes in the colon mucosa reflect imbalance in inflammation and sufficiency of protective function and reparative processes in the mucosal barrier of the large intestine in different type of IBD.
